# Supplementary material for: Incidence, Mortality, and Trends of Prostate Cancer in Mexico from 2000 to 2019: Results from the Global Burden of Disease Study 2019
Source: Cancers (Basel). 2022 Jun 29;14(13):3184. doi: 10.3390/cancers14133184 (PMC9265044; doi:10.3390/cancers14133184)
Supplement: Supplementary file 1 [file cancers-14-03184-s001.zip › cancers-1732230-supplementary.pdf]

cancers MDPI

# Supplementary Material: Incidence, Mortality, and Trends of Prostate Cancer in Mexico from 2000 to 2019: Results from the Global Burden of Disease Study 2019

**Saul A. Beltran-Ontiveros, Martha A. Fernandez-Galindo, Jose M. Moreno-Ortiz, Jose A. Contreras-Gutierrez, Jesus Madueña-Molina, Eliakym Arambula-Meraz, Emir Leal-Leon, Delia M. Becerril-Camacho, Veronica J. Picos-Cardenas, Carla Angulo-Rojo, Diana Z. Velazquez, Francisco Jimenez-Trejo, Francisco Gallardo-Vera and Daniel Diaz**

**Supplementary Table S1.** National and subnational incidence of prostate cancer in Mexico.

| Incidence | Counts (95% UI) | | | Percentage change counts 2010 to 2019 | ASR (per 100,000) |
|---|---|---|---|---|---|
| | 2000 | 2010 | 2019 | | 2019 |
| **National** | 11444 (8883-14016) | 17784 (14346-23397) | 27096 (20602-36016) | 52.4 (24.0-88.0) | 52.3 (40.0-70.1) |
| Aguascalientes | 91 (75-119) | 154 (120-201) | 260 (190-355) | 69.0 (28.9-118.5) | 52.7 (38.3-71.8) |
| Baja California | 288 (171-338) | 471 (316-552) | 844 (568-1154) | 79.4 (34.3-134.4) | 62.3 (42.7-84.5) |
| Baja California Sur | 58 (38-68) | 99 (68-118) | 207 (135-294) | 110.0 (55.1-190.2) | 74.9 (49.7-104.0) |
| Campeche | 69 (57-101) | 119 (99-171) | 165 (119-250) | 39.1 (5.4-82.2) | 42.6 (30.8-64.4) |
| Chiapas | 289 (194-340) | 529 (357-618) | 821 (572-1097) | 55.0 (17.6-102.8) | 41.3 (28.7-55.0) |
| Chihuahua | 377 (254-436) | 537 (363-628) | 837 (521-1162) | 55.8 (18.0-102.1) | 60.4 (37.8-83.2) |
| Coahuila | 292 (212-347) | 423 (336-569) | 665 (479-930) | 57.1 (18.6-107.2) | 52.6 (38.1-73.4) |
| Colima | 74 (52-87) | 122 (91-148) | 206 (135-298) | 69.4 (22.0-141.8) | 65.9 (43.5-94.5) |
| Durango | 180 (139-225) | 251 (195-322) | 381 (272-508) | 51.6 (12.7-97.5) | 48.9 (35.0-65.3) |
| Guanajuato | 484 (399-659) | 783 (657-1107) | 1214 (867-1745) | 55.1 (15.9-114.9) | 51.4 (36.8-73.5) |
| Guerrero | 440 (341-672) | 799 (655-1179) | 989 (770-1424) | 23.8 (0.6-58.4) | 69.4 (54.2-101.6) |
| Hidalgo | 225 (185-313) | 379 (321-553) | 599 (425-901) | 58.1 (18.2-110.1) | 44.5 (31.7-66.8) |
| Jalisco | 913 (619-1049) | 1412 (1000-1662) | 2027 (1400-2702) | 43.5 (6.5-89.5) | 60.6 (41.8-80.1) |
| Mexico | 1003 (844-1401) | 1731 (1478-2546) | 2807 (2071-4226) | 62.2 (26.0-108.6) | 44.6 (33.1-67.5) |
| Mexico City | 1303 (858-1523) | 1636 (1256-2137) | 2529 (1797-3517) | 54.6 (18.0-99.4) | 55.7 (39.9-77.1) |
| Michoacan | 548 (375-655) | 765 (583-970) | 1161 (804-1566) | 51.8 (14.2-99.7) | 53.3 (36.9-72.0) |
| Morelos | 186 (152-248) | 284 (239-403) | 416 (294-595) | 46.3 (7.8-95.4) | 45.8 (32.5-65.8) |
| Nayarit | 153 (102-180) | 272 (183-322) | 412 (259-568) | 51.2 (8.2-107.7) | 71.4 (44.9-98.4) |
| Nuevo Leon | 553 (426-699) | 868 (684-1133) | 1361 (990-1925) | 56.8 (18.5-103.3) | 62.3 (45.6-87.7) |
| Oaxaca | 311 (260-459) | 498 (419-778) | 668 (490-1069) | 34.2 (2.7-76.8) | 33.8 (24.7-54.0) |
| Puebla | 414 (355-597) | 643 (541-982) | 896 (643-1369) | 39.4 (3.8-79.1) | 36.1 (26.1-54.9) |
| Queretaro | 123 (103-167) | 207 (173-295) | 351 (251-524) | 70.1 (27.4-124.3) | 46.3 (33.4-68.7) |
| Quintana Roo | 51 (42-74) | 108 (89-160) | 200 (145-302) | 85.4 (39.1-147.1) | 44.5 (32.6-68.4) |
| San Luis Potosi | 272 (205-343) | 404 (321-528) | 604 (442-850) | 49.4 (12.6-105.6) | 46.5 (34.0-65.3) |
| Sinaloa | 444 (300-513) | 728 (518-857) | 1221 (807-1695) | 67.8 (21.1-128.6) | 85.2 (56.7-118.1) |
| Sonora | 340 (212-397) | 529 (337-619) | 798 (499-1088) | 50.8 (14.9-100.2) | 63.0 (39.7-85.5) |
| Tabasco | 207 (132-242) | 338 (241-396) | 484 (332-644) | 43.1 (8.1-90.0) | 50.2 (34.6-66.6) |
| Tamaulipas | 411 (311-497) | 639 (471-783) | 986 (685-1360) | 54.3 (12.8-103.1) | 67.3 (47.3-92.2) |
| Tlaxcala | 80 (66-132) | 124 (101-207) | 191 (133-312) | 53.9 (13.1-103.4) | 37.3 (26.0-61.0) |
| Veracruz | 890 (622-1040) | 1363 (1033-1661) | 1988 (1451-2610) | 45.8 (11.4-90.2) | 49.9 (36.5-65.6) |
| Yucatan | 191 (159-292) | 293 (245-464) | 410 (288-653) | 39.8 (4.5-80.1) | 41.6 (29.4-66.2) |
| Zacatecas | 183 (148-245) | 277 (228-366) | 397 (284-552) | 43.3 (5.7-93.3) | 53.7 (38.4-75.1) |

ASR = Age-standardized rate.

**Supplementary Table S2.** National and subnational mortality of prostate cancer in Mexico.

| Deaths | Counts (95% UI) | | | Percentage change counts 2010 to 2019 | ASR (per 100,000) |
|---|---|---|---|---|---|
| | 2000 | 2010 | 2019 | | 2019 |
| **National** | 4812 (3795-6138) | 6864 (5674-9075) | 9256 (7077-12678) | 34.8 (13.0-62.0) | 19.4 (14.8-26.7) |
| Aguascalientes | 39 (32-52) | 61 (47-77) | 85 (63-114) | 39.0 (9.7-71.4) | 19.6 (14.6-26.4) |
| Baja California | 103 (64-117) | 147 (103-174) | 228 (153-295) | 55.5 (22.6-92.9) | 21.3 (14.4-27.4) |
| Baja California Sur | 20 (13-23) | 31 (23-37) | 53 (34-73) | 69.9 (31.1-124.9) | 24.2 (16.1-33.0) |
| Campeche | 28 (24-42) | 45 (38-66) | 59 (43-90) | 29.4 (1.2-62.4) | 16.0 (11.8-24.6) |
| Chiapas | 157 (108-197) | 263 (174-301) | 377 (244-488) | 43.4 (15.0-78.1) | 20.8 (13.5-26.6) |
| Chihuahua | 160 (111-183) | 220 (156-266) | 295 (193-392) | 34.2 (5.5-68.0) | 23.7 (15.7-31.3) |
| Coahuila | 114 (88-138) | 156 (126-207) | 217 (160-300) | 39.2 (10.7-74.0) | 19.3 (14.3-26.9) |
| Colima | 31 (22-37) | 47 (34-56) | 69 (44-97) | 46.7 (13.9-98.8) | 25.5 (16.4-35.5) |
| Durango | 79 (61-103) | 109 (87-142) | 136 (98-183) | 25.0 (-2.5-57.3) | 17.6 (12.6-23.7) |
| Guanajuato | 215 (178-289) | 312 (262-447) | 421 (307-603) | 35.0 (3.3-75.3) | 19.1 (14.0-27.5) |
| Guerrero | 215 (165-353) | 354 (297-540) | 378 (302-580) | 6.9 (-10.4-30.2) | 29.8 (23.7-46.9) |
| Hidalgo | 98 (82-151) | 149 (126-222) | 208 (152-329) | 39.4 (9.0-76.7) | 15.9 (11.7-25.3) |
| Jalisco | 397 (275-445) | 537 (385-609) | 701 (489-904) | 30.6 (3.4-62.8) | 22.8 (16.0-29.5) |
| Mexico | 391 (340-558) | 614 (525-914) | 909 (681-1368) | 48.0 (17.6-85.4) | 16.9 (12.6-25.7) |
| Mexico City | 510 (348-593) | 608 (474-800) | 810 (587-1120) | 33.1 (5.3-65.3) | 19.7 (14.3-27.2) |
| Michoacan | 252 (178-301) | 337 (257-440) | 441 (317-588) | 30.9 (3.5-66.4) | 19.6 (14.1-26.1) |
| Morelos | 74 (62-101) | 111 (92-157) | 152 (112-224) | 36.5 (6.9-74.2) | 17.9 (13.2-26.3) |
| Nayarit | 64 (45-75) | 104 (72-120) | 140 (89-185) | 34.6 (4.7-77.3) | 24.6 (15.5-32.4) |
| Nuevo Leon | 181 (143-233) | 263 (210-353) | 364 (269-508) | 38.8 (7.8-71.2) | 18.8 (14.0-26.0) |
| Oaxaca | 160 (134-246) | 241 (199-391) | 309 (227-497) | 28.1 (3.1-61.7) | 15.1 (11.0-24.3) |
| Puebla | 211 (179-305) | 290 (244-447) | 373 (277-583) | 28.5 (2.3-60.3) | 15.7 (11.6-24.6) |
| Queretaro | 51 (43-70) | 79 (66-111) | 118 (88-173) | 50.4 (18.7-89.5) | 18.4 (13.7-26.5) |
| Quintana Roo | 18 (15-27) | 36 (30-55) | 61 (45-95) | 68.7 (31.9-115.7) | 17.6 (13.2-27.8) |
| San Luis Potosi | 126 (97-165) | 177 (140-238) | 235 (173-329) | 32.7 (3.2-72.6) | 18.0 (13.2-25.1) |
| Sinaloa | 157 (107-178) | 221 (162-260) | 316 (216-426) | 43.1 (9.1-84.7) | 23.1 (15.9-31.0) |
| Sonora | 127 (80-143) | 185 (121-211) | 244 (161-318) | 32.1 (2.5-64.5) | 22.3 (14.9-29.1) |
| Tabasco | 86 (55-98) | 129 (94-148) | 173 (119-222) | 34.0 (4.8-66.0) | 19.6 (13.5-25.2) |
| Tamaulipas | 135 (109-174) | 207 (155-256) | 277 (199-372) | 33.8 (5.4-65.7) | 20.2 (14.5-27.0) |
| Tlaxcala | 36 (30-61) | 53 (42-90) | 70 (50-119) | 33.1 (5.6-71.2) | 14.2 (10.2-24.2) |
| Veracruz | 402 (295-499) | 534 (412-678) | 729 (531-980) | 36.6 (10.3-72.4) | 19.1 (13.9-25.7) |
| Yucatan | 84 (71-131) | 118 (101-186) | 152 (112-241) | 28.4 (2.8-60.5) | 16.8 (12.5-26.9) |
| Zacatecas | 87 (70-118) | 127 (102-177) | 156 (115-214) | 23.0 (-4.3-61.0) | 20.3 (14.9-27.9) |

ASR = Age-standardized rate.

**Supplementary Table S3.** National and subnational case fatality rate due to prostate cancer in Mexico during 2019.

| Level | Death counts | Incidence counts | CFR (%) |
|---|---|---|---|
| National | 9256 | 27096 | 34.16 |
| Aguascalientes | 85 | 260 | 32.69 |
| Baja California | 228 | 844 | 27.01 |
| Baja California Sur | 53 | 207 | 25.60 |
| Campeche | 59 | 165 | 35.76 |
| Chiapas | 377 | 821 | 45.92 |
| Chihuahua | 295 | 837 | 35.24 |
| Coahuila | 217 | 665 | 32.63 |
| Colima | 69 | 206 | 33.50 |
| Durango | 136 | 381 | 35.70 |
| Guanajuato | 421 | 1214 | 34.68 |
| Guerrero | 378 | 989 | 38.22 |
| Hidalgo | 208 | 599 | 34.72 |
| Jalisco | 701 | 2027 | 34.58 |

| | | | |
|---|---|---|---|
| Mexico | 909 | 2807 | 32.38 |
| Mexico City | 810 | 2529 | 32.03 |
| Michoacan | 441 | 1161 | 37.98 |
| Morelos | 152 | 416 | 36.54 |
| Nayarit | 140 | 412 | 33.98 |
| Nuevo Leon | 364 | 1361 | 26.75 |
| Oaxaca | 309 | 668 | 46.26 |
| Puebla | 373 | 896 | 41.63 |
| Queretaro | 118 | 351 | 33.62 |
| Quintana Roo | 61 | 200 | 30.50 |
| San Luis Potosi | 235 | 604 | 38.91 |
| Sinaloa | 316 | 1221 | 25.88 |
| Sonora | 244 | 798 | 30.58 |
| Tabasco | 173 | 484 | 35.74 |
| Tamaulipas | 277 | 986 | 28.09 |
| Tlaxcala | 70 | 191 | 36.65 |
| Veracruz | 729 | 1988 | 36.67 |
| Yucatan | 152 | 410 | 37.07 |
| Zacatecas | 156 | 397 | 39.29 |

CFR: Case fatality rate = Mortality / Incidence x 100.

**Supplementary Table S4.** Incidence and mortality of prostate cancer by age group in Mexico in 2019 and percentage changes from 2010 to 2019.

| Age group | Rate (95% UI) per 100,000 | | | |
|---|---|---|---|---|
| | **Incidence** | **% change 2010-2019** | **Deaths** | **% change 2010-2019** |
| **40-44** | 2.4 (1.7 to 3.3) | 19.2 (-9.1 to 58.3) | 0.3 (0.2 to 0.4) | 2.4 (-19.7 to 33.0) |
| **45-49** | 8.9 (6.2 to 12.4) | 15.7 (-12.5 to 51.1) | 1.1 (0.8 to 1.6) | -0.9 (-24.1 to 28.2) |
| **50-54** | 29.9 (20.6 to 41.5) | 12.7 (-15.0 to 46.9) | 3.5 (2.3 to 4.8) | -3.6 (-26.2 to 25.7) |
| **55-59** | 82.6 (57.1 to 112.1) | 17.5 (-11.0 to 53.7) | 9.9 (6.7 to 13.8) | 0.5 (-22.3 to 31.0) |
| **60-64** | 192.1 (139.5 to 261.2) | 16.4 (-10.2 to 50.6) | 28.1 (19.9 to 38.1) | 0.2 (-21.4 to 28.1) |
| **65-69** | 325.0 (237.3 to 434.4) | 14.9 (-9.2 to 45.7) | 58.6 (41.3 to 80.0) | -0.9 (-20.8 to 24.9) |
| **70-74** | 440.4 (331.5 to 611.0) | 9.8 (-11.5 to 35.8) | 114.1 (84.5 to 155.6) | -4.1 (-22.4 to 19.6) |
| **75-79** | 529.6 (410.1 to 721.0) | 8.4 (-11.5 to 31.9) | 221.0 (169.7 to 303.4) | -3.4 (-20.2 to 17.1) |
| **80-84** | 531.3 (410.1 to 716.8) | 5.2 (-12.2 to 25.9) | 353.2 (262.1 to 477.1) | -3.0 (-19.1 to 15.6) |
| **85-89** | 564.0 (431.9 to 821.5) | 2.8 (-10.4 to 18.4) | 550.2 (426.0 to 787.2) | -1.9 (-15.0 to 12.5) |
| **90-94** | 523.4 (382.8 to 714.9) | 12.3 (-5.9 to 38.4) | 680.4 (488.3 to 932.4) | 8.8 (-9.2 to 36.0) |
| **>95** | 535.8 (382.7 to 763.7) | 26.5 (11.3 to 45.4) | 818.0 (569.6 to 1172.2) | 22.9 (7.2 to 40.9) |

**Supplementary Table S5.** Estimates of Average Annual Percent Change and Annual Percent Change of the age-standardized rate of incidence and deaths (per 100,000 people) due to prostate cancer in Mexico.

| Estimate | Metric | | | | |
|---|---|---|---|---|---|
| | **Range** | **Period** | **AAPC (95% CI)** | **Test Statistic (t)** | **Prob > \|t\|** |
| | Full range | 2000 - 2019 | 0.9 (0.7 to 1.2)* | 7.0 | < 0.001 |
| **Incidence** | **Segment** | **Period** | **APC (95% CI)** | **Test Statistic (t)** | **Prob > \|t\|** |
| | 1 | 2000 - 2003 | 1.4 (0.2 to 2.6)* | 2.6 | 0.022 |
| | 2 | 2003 - 2016 | 0.4 (0.3 to 0.6)* | 6.6 | < 0.001 |
| | 3 | 2016 - 2019 | 2.7 (1.4 to 4.0)* | 4.5 | 0.001 |
| | **Range** | **Period** | **AAPC (95% CI)** | **Test Statistic (t)** | **Prob > \|t\|** |
| | Full range | 2000 - 2019 | -0.4 (-0.6 to -0.3)* | -5.2 | < 0.001 |
| **Deaths** | **Segment** | **Period** | **APC (95% CI)** | **Test Statistic (t)** | **Prob > \|t\|** |
| | 1 | 2000 - 2007 | -0.8 (-1.1 to -0.4)* | -4.5 | < 0.001 |
| | 2 | 2007 - 2019 | -0.2 (-0.4 to -0.0)* | -2.7 | 0.015 |

AAPC: Average Annual Percent Change; APC: Annual Percent Change.
* Indicates that the APC/AAPC is significantly different from zero at $p < 0.05$.

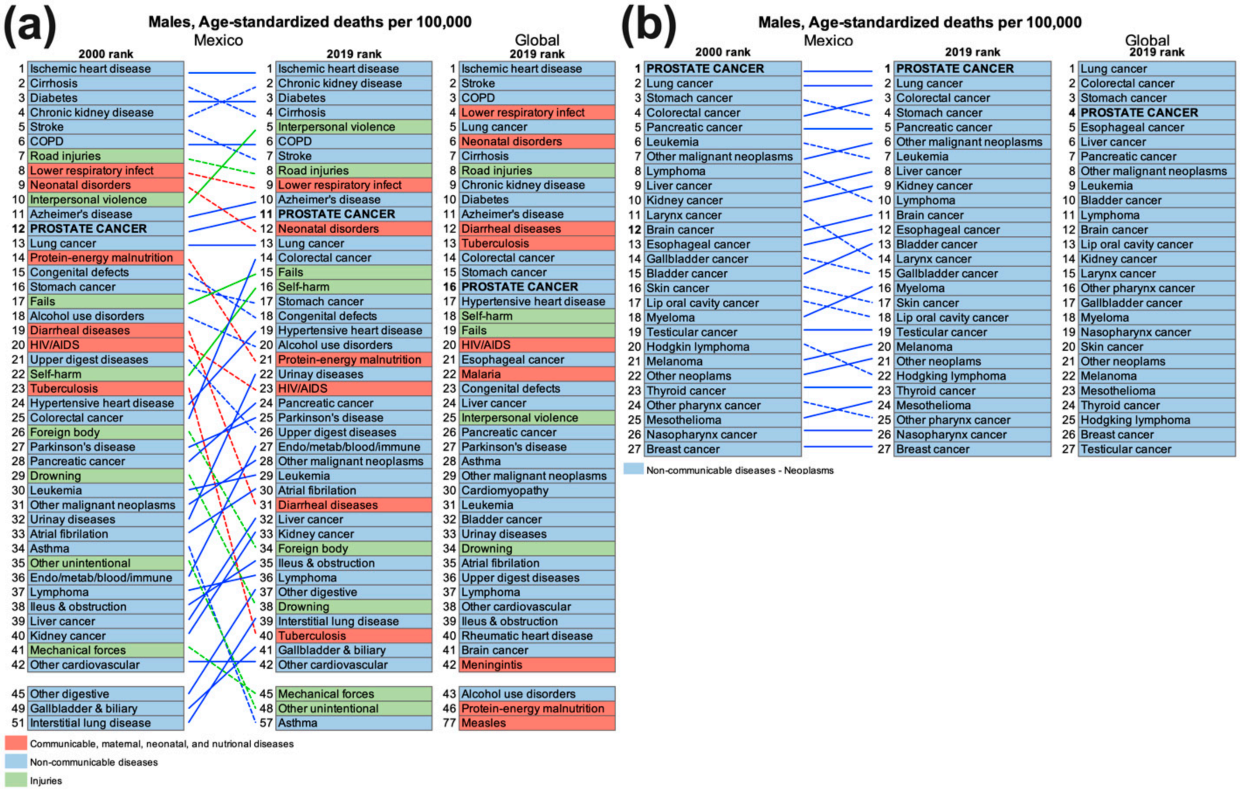


**Supplementary Figure S1.** Global and local ranking of (**a**) the 45 diseases and injuries included in the GBD hierarchy level 3; and (**b**) the 27 groups of neoplasms.

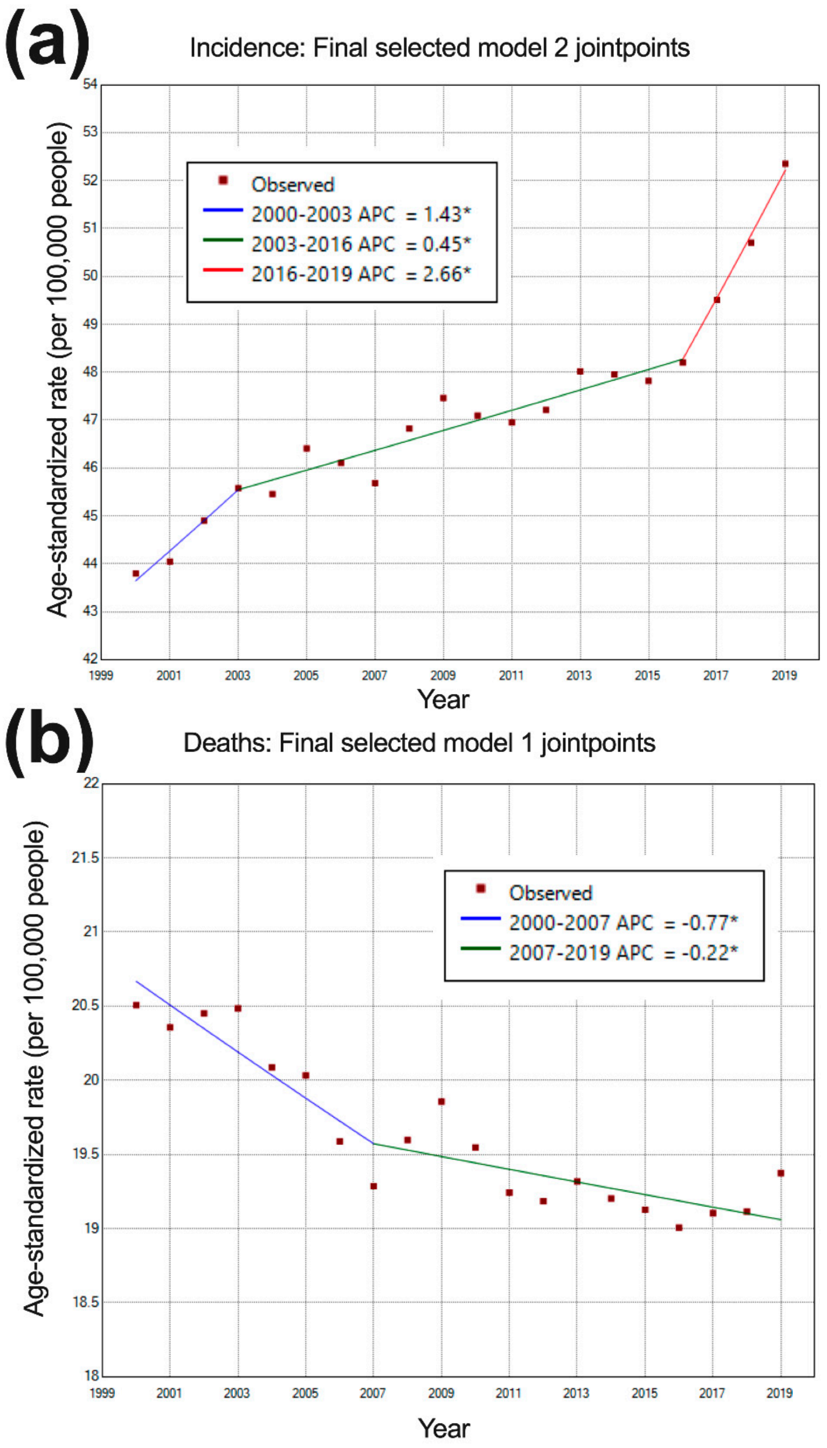


* Indicates that the Annual Percent Change (APC) is significantly different from zero at $p < 0.05$

**Supplementary Figure S2.** Results from jointpoint regression analysis of (**a**) incidence and (**b**) deaths of prostate cancer in Mexico from 2000 to 2019.

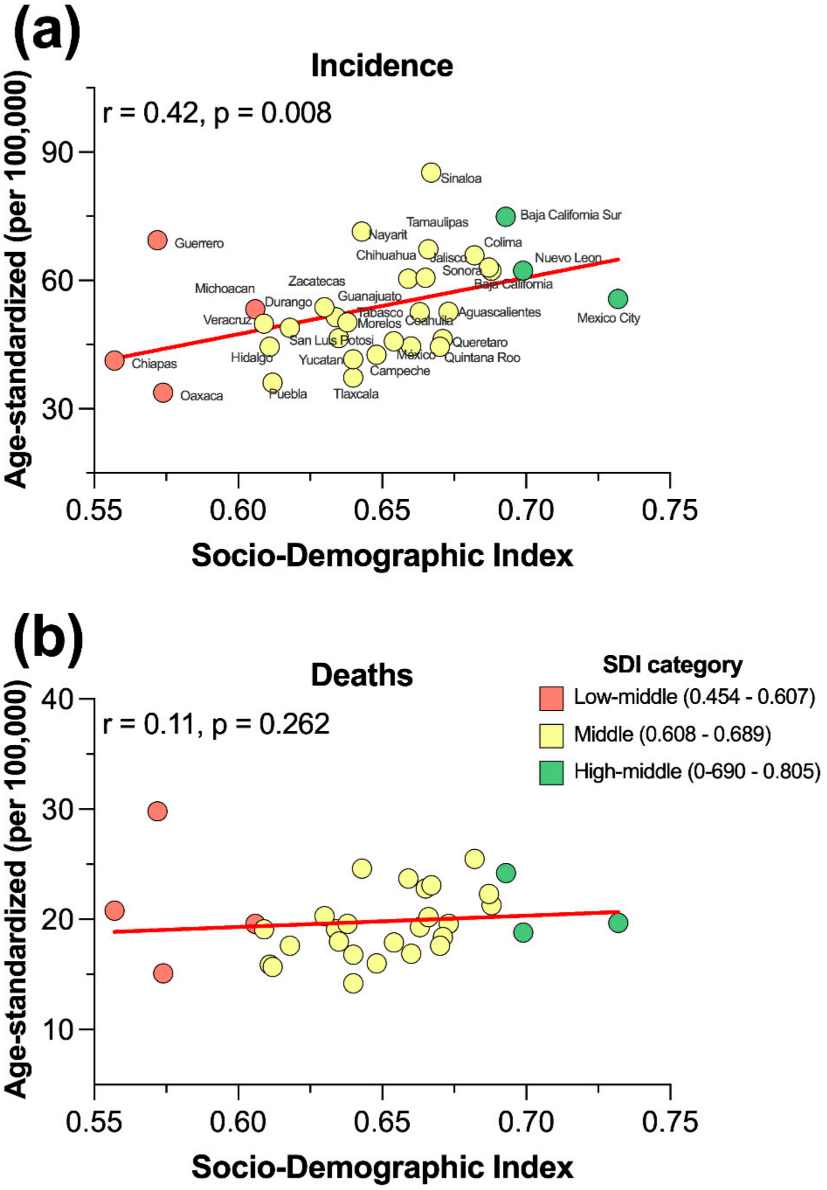


**Supplementary Figure S3.** Association of the sociodemographic index (SDI) and age-standardized (**a**) incidence and (**b**) death counts per 100,000 people at the subnational level in Mexico in 2019.
